# Effect of Restriction of Fluoroquinolone Antibiotics on *Clostridioides difficile* Infections in the University Hospital Hradec Králové

**DOI:** 10.3390/antibiotics10050519

**Published:** 2021-05-02

**Authors:** Kristýna Vaverková, Martin Kracík, Lenka Ryšková, Pavla Paterová, Rudolf Kukla, Lenka Hobzová, Roman Špánek, Helena Žemličková

**Affiliations:** 1 Department of Clinical Microbiology, University Hospital and Faculty of Medicine in Hradec Kralove, Charles University, 50005 Hradec Kralove, Czech Republic; kristyna.vaverkova@fnhk.cz (K.V.); martin.kracik@nemlib.cz (M.K.); lenka.ryskova@fnhk.cz (L.R.); pavla.paterova@fnhk.cz (P.P.); rudolf.kukla@fnhk.cz (R.K.); 2 Department of Clinical Microbiology and Imunology, Regional Hospital Liberec, 46001 Liberec, Czech Republic; 3 Hospital Hygiene Department, University Hospital Hradec Kralove, 50005 Hradec Kralove, Czech Republic; lenka.hobzova@fnhk.cz; 4 Faculty of Mechatronics, Informatics and Interdisciplinary Studies, Technical University of Liberec, 46001 Liberec, Czech Republic; roman.spanek@tul.cz; 5 National Reference Laboratory for Antibiotics, Centre for Epidemiology and Microbiology, National Institute of Public Health, 10000 Prague, Czech Republic; 6 Department of Microbiology, 3rd Faculty of Medicine Charles University, University Hospital Kralovske Vinohrady and National Institute of Public Health, 10000 Prague, Czech Republic

**Keywords:** *Clostridioides difficile*, *C. difficile* infections, antibiotic stewardship, capillary electrophoresis ribotyping, fluoroquinolones

## Abstract

*Clostridioides difficile* is the most common pathogen responsible for hospital-acquired diarrhea. This complication of antibiotic treatment mainly endangers the health of elder patients. Preventing the development of *C. difficile* infections (CDI) is still a challenge that needs to be addressed. In our study, the results of 872 *C. difficile* positive stool samples were used to describe the epidemiological situation affected by a change in the prescription of fluoroquinolone antibiotics. In a total, 93 of strains were typed by polymerase chain reaction (PCR) and capillary gel electrophoresis. Between years 2014 and 2018 the decline in the fluoroquinolones consumption was 69.3 defined daily dose (DDD) per 1000 patient-days (from 103.3 to 34.0), in same period CDI incidence declined by 1.3 cases per 10,000 patient-bed days (from 5.6 to 4.3). Results of epidemiologic and statistical analysis shows that decline in fluoroquinolones consumption has significant influence on CDI incidence and prevalence of hypervirulent strains. In the University Hospital Hradec Králové properly managed antibiotic stewardship policy has reduced CDI incidence by 23.2% and lowered rate of hypervirulent ribotypes 001 and 176.

## 1. Introduction

*Clostridium difficile*, recently reclassified as *Clostridioides difficile* [1] is the most common pathogen responsible for hospital-acquired diarrhea which is associated especially with previous antibiotic therapy. In particular, elder patients over 65 years with simultaneous presence of other comorbidities are endangered by more serious course of *C. difficile* infection (CDI) manifesting as pseudomembranous colitis, intestinal perforation, and septic shock leading to the patient death [2]. However, CDI represent not only a medical but also an epidemiological challenge. In Europe, CDI incidence showed an increasing trend [3,4] and in the Czech republic the epidemiological situation is also undesirable due to increasing occurrence of epidemic strains, especially PCR ribotype (RT) 176 and 001 that participate significantly in the severe courses of CDI [5,6]. A common feature of these two ribotypes is their multidrug resistance, notably a natural resistance to fluoroquinolone antibiotics [7,8]. This is the reason why the fluoroquinolones are one of the most hazardous group of antibiotics contributing to the spread of these highly virulent epidemic strains [8]. In particular, a hypervirulent RT 176, genetically closely related to RT 027, which has spread worldwide, is a serious threat due to its hypertoxigenicity (caused by mutation in *tcdC* gene leading to hyperproduction of toxin B) and production of binary toxin [7,9]. CDI caused by this strain manifest with severe course of infection, more frequent recurrences and higher mortality [10].

In the University Hospital Hradec Králové, a control over prescription of certain antibiotics falls within competence of the Antibiotic Centre, Department of Clinical Microbiology. There has been an effort of the Antibiotic Centre to reduce an antimicrobial consumption from the year 2010 by creating a list of antibiotics requiring their authorization. Concerning fluoroquinolone antibiotics, at first, the parenteral forms were restricted. In 2012, the peroral forms of levofloxacin and moxifloxacin were included to the list and in 2015 norfloxacin was included as well. In 2017, all forms of all registered fluoroquinolone antibiotics were bound to the Antibiotic center’s authorization. This measure was implemented on the basis of decision of European Medicines Agency (EMA) to reevaluate the use of quinolones and fluoroquinolones due to their disabling and potentially permanent side effects (London, UK). Another reason for this measure was a long-term strategy of the Subcommittee for Antibiotic Policy of the Czech Medical Society of Jan Evangelista Purkyně (SKAP CLS JEP, Prague, Czech Republic) which formulated the principles of national antibiotic policy including restriction of fluoroquinolones use due to their rapidly growing resistance. According to their official recommendations, the fluoroquinolones are reserve antibiotics, indicated in situations only as a secondary choice, and when their efficacy is verified by susceptibility testing [11]. In accordance with EMA reevaluation and the SKAP CLS JEP official recommendation, the Antibiotic Centre formulated the empirical antibiotic therapy guidelines, reducing the use of fluoroquinolones. Concurrently with gradual limitation in fluoroquinolone use, a decline in amount of CDI was described; therefore, an impact of fluoroquinolone restriction to the CDI incidence has begun to be actively monitored.

## 2. Results

In total, between years 2014 and 2018, 872 cases of CDI (demography in Supplementary Materials Table S1) were identified among 7434 tested samples (4413 patients) in the University Hospital Hradec Králové. The data obtained from the Hospital Hygiene Department reported a decrease in CDI incidence by 23.2% from 5.6 cases per 10,000 patient-bed days in 2014 to 4.3 cases per 10,000 patient-bed days in 2018. Thanks to the antibiotic stewardship intervention, during the monitored years, the consumption of fluoroquinolone antibiotics (J01M) decreased by 67.1% from 103.3 DDD per 1000 patient-days in 2014 to 34.0 DDD per 1000 patient-days in 2018.

**Figure 1:**
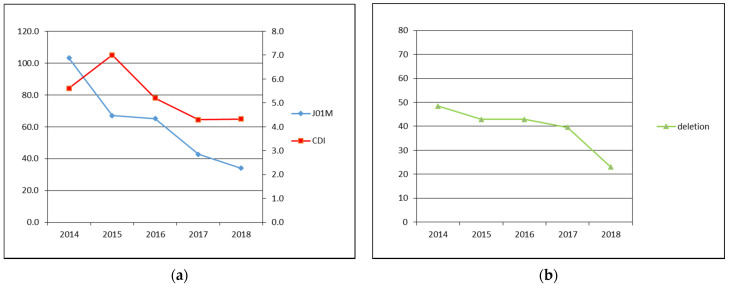
(**a**) Primary axis: J01M—fluoroquinolone antibiotics consumption in DDD per 1000 patient-days, secondary axis: CDI—CDI incidence in cases per 10,000 patient-bed days; (**b**) deletion—prevalence of *tcdC* gene deletion identified by GeneXpert in %.

The data about total number of examined patients and patients diagnosed with the CDI, the CDI incidence, prevalence of *tcdC* gene deletion and fluoroquinolones consumption are shown in Table 1 and Figure 1.

### 2.1. Fluoroquinolone Antibiotics Consumption, CDI Incidence, Prevalence of tcdC Gene Deletion

**Figure 2:**
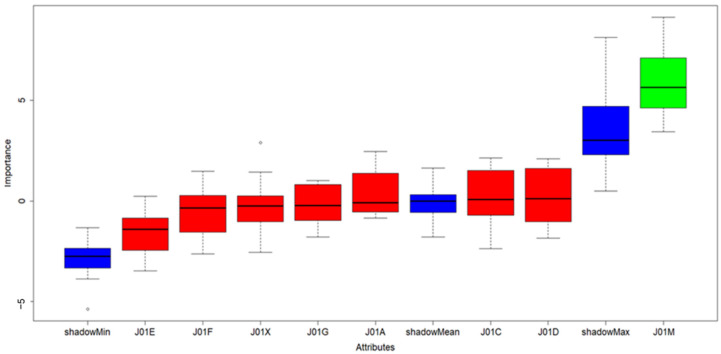
Results of Boruta algorithm; color legend: blue boxes: shadow features added by Boruta algorithm used for evaluation of significant and insignificant variables in scope. Green box: significant/important properties, Red boxes: insignificant/redundant properties in dataset.

The causative antibiotics (ATBs) influence to occurrence of CDI was tested on monthly ATBs consumption (Table S2) by feature selection approach, particularly Boruta available as R software package. The Boruta results clearly confirmed that only fluoroquinolones (shown in green in Figure 2) had significant influence on CDI and their regulation is therefore crucial for CDI prevention.

### 2.2. Significance of ATB Groups to Occurrence of CDI

All *C. difficile* positive samples were tested by GeneXpert real-time PCR system to detect *tcdC* gene deletion. During this year, the prevalence of *tcdC* gene deletion decreased from 48.4% (*n* = 90) in 2014 to 23.0% (*n* = 32) in 2018. All viable strains (*n* = 93; 66.9%) of *C. difficile* isolated in 2018 (*n* = 139) were later PCR ribotyped. The prevalent ribotypes (RTs) was 001 (*n* = 18, 19.4%), 014 (*n* = 12, 12.9%), 078 (*n* = 7, 7.5%), 176 (*n* = 6, 6.5%), and 020 (*n* = 6, 6.5%). The occurrence of other 25 RTs (*n* = 38, 40.9%) did not exceed 3.0% (Table S3). In 6 strains ribotyping repeatedly failed.

## 3. Discussion

Our hypothesis was that fluoroquinolone restriction leads to decline in CDI incidence and to higher diversity of ribotypes (lower prevalence of hypervirulent strains like RT001, RT027, or RT176). This hypothesis is supported by several sources.

The study performed in England describe the decline in total CDI incidence driven by reduction of fluoroquinolone-resistant strains. The incidence of fluoroquinolone-susceptible strains remained constant between years 2005 and 2010 when fluoroquinolone prescribing had been restricted. Incidence of CDI caused by fluoroquinolone-susceptible strains was unaffected by changes in fluoroquinolone use or other national policy measures, such as restricted cephalosporin prescribing and enhanced infection control interventions. The restriction of fluoroquinolones played the most significant factor in their success. Consequently, the antimicrobial stewardship was found to play a role in the control of CDI, as selective advantage specific to resistant isolates is suppressed when the antimicrobials are restricted [12].

Another study, showing a similar conclusion was conducted in Scotland. This study demonstrated that decline in rates of CDI between years 1997–2012 is primarily due to decline of the multidrug-resistant *C. difficile* strains (RT001 and RT027). This decline was also connected with restricted use of particular antibiotic classes, including the fluoroquinolone antibiotics, both in hospital and ambulatory care. CDI prevalence dropped by 68% in hospitals and 45% in the community. In this study were also used other interventions methods like limiting indications for macrolide prescriptions, introduction of alcohol-based hand sanitizer, a national hand-hygiene campaign, national auditing and inspections of hospital environment cleanliness, and reminders to reduce inappropriate use of proton-pump inhibitors [13].

Our data support our hypothesis and showed a similar trend demonstrated in mentioned studies. Statistical analysis confirmed that decline in fluoroquinolones consumption during monitored years has causative influence to CDI incidence. The CDI incidence dropped by 23.2% (from 5.6 in 2014 to 4.3 in 2018 of cases per 10,000 patient-bed days) during five years.

On the other hand, a downward trend of CDI incidence in our study was also observed in data from whole Czech Republic. The mean CDI incidence between 2014 and 2017 was 6.1 to 4.5 CDI cases per 10,000 patient-bed days [14]. Compare to Czech data European mean CDI incidence is constant. In 2010, a hospital-based survey determined mean CDI incidence at 4.1 cases per 10,000 patient-bed days [3]. In first Annual Epidemiological Report for 2016 of European Centre for Disease Prevention and Control (ECDC), the mean CDI incidence was 3.98 cases per 10,000 patient-bed days [15]. This show that downward trend in CDI incidence could be specific to the Czech Republic not only to the University Hospital Hradec Králové.

Some studies showed that decline of CDI incidence is mainly caused by decrease of hypervirulent ribotypes which are resistant to fluoroquinolones [12,13]. In our study, the occurrence of hypervirulent strains was monitored by detecting a deletion in the *tcdC* gene using commercial GenExpert system. The prevalence of strains with this specific deletion declined in correlation with decrease of CDI incidence, and use of fluoroquinolones.

The Czech national survey of CDI including genotyping of *C. difficile* strains ongoing in 2017 identified RT 001 (33.5%) and RT 176 (11.6%) as the most frequent ribotypes [14]. Importantly, the *C. difficile* isolates belonging to RTs 001 and 176 were also associated with reduced susceptibility to moxifloxacin. In our study, the most prevalent ribotypes (RTs 001, 014, 078, 176, and 020) take 52.8% of all detected RTs, but the proportion of RT 001 (19.4%) and RT 176 (6.5%) was much lower.

The population snapshot of strains from CDI cases in 2018 revealed lower rate of hypervirulent RTs 001 and 176 in University hospital in Hradec Králové in comparison with national study but CDI incidence was quite similar (4.3 vs. 4.5 of cases per 10,000 patient-bed days). The tentative explanation of this discrepancy is gradual implementation of antibiotic stewardship policy in hospital.

The present study has several limitations. Typing data consist only on larger part (66.9%) of isolated *C. difficile* strains from 2018. Data from previous years are not available. So connections between lower rate of hypervirulent RTs 001 and 176 and lower fluoroquinolone consumption are based on information from literature. The occurrence of RTs 001 and 176 may have been the same in previous years, but compared to national data, this possibility is less likely. By literature the decline in CDI incidence is connected with decline of fluoroquinolone-resistant strains. In the University Hospital Hradec Králové routine susceptibility testing on fluroquinolone is not established. The hospital tests only clinically relevant antimicrobials. Therefore, we derive decline of resistant strains from prevalence of strains with *tcdC* gene deletion.

## 4. Materials and Methods

A CDI case was defined as a patient suffering from diarrhea with laboratory confirmation of *C. difficile*. In the University hospital Hradec Králové laboratory confirmation was performed in two steps. First, C. Diff Quik Chek Complete^®^ EIA (TechLab, Blacksburg, VA, USA) was used for rapid simultaneous detection of GDH and toxin A/B. Then, in case of positivity (positive GDH with positive or negative toxin A/B), testing proceeded with anaerobic cultivation on selective medium (followed by susceptibility testing of therapeutic antibiotics—metronidazole and vancomycin) and detection of toxin-producing *C. difficile* strains using commercial kit Xpert^®^ *C. difficile* (Cepheid, Sunnyvale, CA, USA) on GeneXpert system for real-time PCR. This kit can detect toxin A and B, binary toxin and deletion of *tcdC* gene (*tcdC*Δ117). Deletion disrupt *TcdC* negative regulation of toxins genes and caused toxins overproduction of hypervirulent strains which include fluoroquinoline resistant strains of RT 027/NAP1/BI (toxinotype III). Ribotyping of cultured *C. difficile* strains by capillary gel electrophoresis-based PCR [16] is recommended for epidemiological situation monitoring. In 2018, DNA isolation from 93 strains was performed using PathogenFree DNA Isolation Kit (GeneProof, Czechia). Isolated DNA was used for PCR reaction: 25 µL Hotstar Mastermix (Qiagen, Netherlands), 10 µL primer mix (0.3 pmol/µL each), forward primer: FAM-16S for (5′-GTGCGGCTGGATCACCTCCT-3′), reverse primer 23S rev (5′-CCCTGCACCCTTAATAACTTGACC-3′), 13 µL nuclease free water, and 2 µL sample DNA. Cycling program was activation 15 min at 95 °C, 30 cycles of denaturation 1 min at 94 °C, annealing 1 min at 60 °C, and elongation 1 min at 72 °C, final elongation step for 30 min at 72 °C. PCR fragments were analysed in an ABI310 genetic analyzer (Thermofisher, Waltham, MA, USA) on 50 cm capillary loaded with a POP4 polymer (Thermofisher, Waltham, MA, USA). As a size standard was used LIZ600 (Thermofisher, Waltham, MA, USA). The size of each peak was determined using GeneMapper software (Thermofisher, Waltham, MA, USA). Profiles gained by capillary gel electrophoresis were uploaded to the WEBRIBO database.

The CDI incidence is expressed as a number of cases per 10,000 patient-bed days and it includes data provided by Hospital Hygiene Department from the years 2014–2018 consisting of non-duplicated positive CDI results without recurrences.

Structured antimicrobial consumption is periodically monitored and a comprehensive review is published annually by the Antibiotic Centre. Antimicrobials are classified according to the Anatomical Therapeutic Chemical (ATC) code and their consumption in Defined Daily Dose (DDD) was calculated according to the actual version of the ATC/DDD index from WHO collaborating Centre for Drug Statistics methodology (Oslo, Norway). Antimicrobial consumption is expressed as a number of DDD per 1000 patient-days and data included in this study consist of average fluoroquinolone consumption from all departments of the University Hospital Hradec Králové over the years 2014–2018.

Statistical analyses were performed in RStudio [17] and R software version 4.0.2 [18]. To evaluate significance of ATBs and their relationship to CDI a well-known and established statistical approach called feature selection was used. Feature selection is a fundamental step in many machine learning pipelines allowing to distinguish between important variables (those having significant impact of a property in scope) from the others (non-important ones). The significance of all ATBs in scope was tested by the Boruta Package [19], built on the Random Forest classification algorithm. Basically Boruta tries to capture all the important, significant features one might have in data set with respect to an outcome variable (CDI in our case). All values (absolute monthly ATBs consumption and number of CDI cases) in dataset were log-transformed prior analyses to achieve normality.

In response to the hospital strategy requiring reduction in use of fluoroquinolone antibiotics, a hospital antibiotic stewardship team comprised of clinical microbiologists and pharmacists, epidemiologist, intensivist, hemato-oncologist, and infectious disease specialist initiated a mixed persuasive and restrictive antibiotic stewardship intervention. Persuasive elements included empirical antibiotic therapy guidelines minimizing use of fluoroquinolone antibiotics, feedback on prescribing and ward-based auditing. Restrictive aspects included fluoroquinolone requiring authorization from a clinical microbiologist and stop-orders (a release of antibiotic only for 3 days of therapy).

## 5. Conclusions

In addition to commonly implemented anti-epidemic measures, the restriction in fluoroquinolones prescribing has proved to be an important element in effort to reduce the CDI incidence, therefore an implementation of antibiotic stewardship should be the cornerstone of the CDI control programme.

## Figures and Tables

**Table 1:** Overview of CDI diagnostics and ATB consumption during years 2014 to 2018.

	2014	2015	2016	2017	2018
Samples ^1^	1527	1617	1435	1354	1501
CDI ^2^	186	233	175	139	139
CDI incidence ^3^	5.6	7.0	5.2	4.3	4.3
Deletion ^4^	48.4 (90)	42.9 (100)	42.9 (75)	39.6 (55)	23.0 (32)
J01M ^5^	103.3	67.1	65.1	42.8	34.0

^1^ Number of tested samples, ^2^ number of confirm CDI, ^3^ CDI incidence in cases per 10,000 patient-bed days, ^4^ prevalence of *tcdC* gene deletion in % (absolute) and ^5^ consumption of fluoroquinolone antibiotics (J01M) in DDD per 1000 patient-days.

## Data Availability

The data presented in this study are available on request from the corresponding author.

## Supplementary Materials

The following are available online at https://www.mdpi.com/article/10.3390/antibiotics10050519/s1, Table S1: Demography data of 872 CDI positive patients. Table S2: Monthly ATB consumption by ATC group (DDD), number of CDI cases and patient-days. Table S3: Ribotypes and phenotypes (C.diff QuikChek based) of the 93 *C. difficile* isolates examined in 2018.
